# Glucocorticoid receptors involved in ginsenoside compound K ameliorate adjuvant arthritis by inhibiting the glycolysis of fibroblast-like synoviocytes via the NF-κB/HIF-1α pathway

**DOI:** 10.1080/13880209.2023.2241512

**Published:** 2023-08-09

**Authors:** Yating Wang, Xiurong Bao, Hao Xian, Fang Wei, Yining Song, Siyu Zhao, Yujie Zhang, Yumeng Wang, Ying Wang

**Affiliations:** a School of Pharmacy, Bengbu Medical College, Bengbu, Anhui, P.R. China; b Anhui Engineering Technology Research Center of Biochemical Pharmaceutical, Bengbu, Anhui, P.R. China

**Keywords:** Inflammation, nuclear factor κB, hypoxia-inducible factor-1α

## Abstract

**Context:**

Ginsenoside metabolite compound K (CK) is an active metabolite produced by ginsenosides *in vivo* that has an anti-arthritic effect related to the glucocorticoid receptor (GR). However, the potential mechanisms of CK remain unclear.

**Objective:**

This study explores the role and potential mechanisms of CK *in vivo* and *in vitro*.

**Materials and methods:**

Adjuvant arthritis (AA) model was induced in Sprague-Dawley (SD) rats; the rats were randomly divided into four groups (*n* = 10): normal, AA, CK (80 mg/kg), and dexamethasone (Dex) group (1 mg/kg). From day 15, rats were treated with CK (once a day, i.g.) and Dex (once every 3 days, i.p.) for 18 days. To further verify the mechanism of CK, fibroblast-like synoviocytes (FLS) were stimulated by tumour necrosis factor α (TNF-α) to establish an inflammatory model *in vitro*.

**Results:**

CK (80 mg/kg) reduced paw swelling (52%) and arthritis global assessment (31%) compared to that in AA rats. In addition, CK (80 mg/kg) suppressed GLUT1 (38%), HK2 (50%), and PKM2 (56%) levels compared with those in AA FLS. However, the effects of CK (30 μM) on these events were weakened or enhanced after GR knockdown or overexpression in FLS stimulated by TNF-α (30 ng/mL). CK (80 mg/kg) also downregulated the expression of P65 (61%), p-IκB (92%), and HIF-1α (59%).

**Discussion and conclusions:**

The inhibition of CK on glycolysis and the NF-κB/HIF-1α pathway is potentially mediated through activating GR. These findings provide experimental evidence for elucidating the molecular mechanism of CK in treating rheumatoid arthritis (RA).

## Introduction

Rheumatoid arthritis (RA) is characterized by abnormal hyperplasia of the synovial tissue in multiple joints, which eventually leads to joint deformity and functional disability (Damerau and Gaber 2020). A satisfactory treatment for RA is not yet known because of its unclear pathogenesis. However, previous studies have indicated that abnormal proliferation of synovial cells and a large extent of inflammatory cell infiltration could cause hypoxia in the RA articular cavity (Quiñonez-Flores et al. 2016). Moreover, in hypoxic environments, rapidly proliferating cells generally prefer the glycolytic pathway to oxidative phosphorylation to sustain their energy supply. Enhanced glycolytic activity can lead to an increase in lactic acid production in synovial fluid, further promoting RA progression (Falconer et al. 2018; Fearon et al. 2019). Therefore, cell metabolism may provide a novel perspective on the pathogenesis of RA. This has been further supported by metabolomic investigations in RA patients (Luan et al. 2021).

As key effector cells and targets in RA treatment, fibroblast-like synoviocytes (FLS) play an important role in the occurrence and promotion of inflammation (Bartok and Firestein 2010; Filer 2013). Recently, changes observed in the metabolic microenvironment of FLS were closely associated with RA progression, particularly the upregulation of glycolysis (Masoumi et al. 2020). The 2-deoxy glucose and 3-bromopyruvate, both glycolytic inhibitors, block the secretion and further diffusion of inflammatory cytokines, significantly alleviating the symptoms of arthritis (Garcia-Carbonell et al. 2016; Wang et al. 2020). As an inhibitor of Janus kinase, tofacitinib can also effectively downregulate the expression of key glycolytic enzymes in RA FLS and treat RA through metabolic reprogramming (McGarry et al. 2018). Therefore, understanding the interactions between and mechanisms involved in cellular metabolism and FLS, may offer novel therapies and directions for RA.

The nuclear factor kappa-B (NF-κB) is crucial for the regulation of cell proliferation, apoptosis, immunity, and inflammatory processes (Hayden and Ghosh 2012; Taniguchi and Karin 2018). Notably, activated NF-κB leads to metabolic reprogramming of aerobic glycolysis, whereas hypoxia in the RA articular cavity is a key contributor in inducing NF-κB activation. The expression of hypoxia-inducible factor-1α (HIF-1α), a nuclear transcription factor that regulates oxygen balance in cells, increases under hypoxia. HIF-1α is reported to increase the expression of glucose transporter and glycolytic enzyme genes to enhance glycolysis and help cells adapt to energy deprivation during hypoxia (Mathupala et al. 2001; Luo et al. 2011; Masoud and Li 2015). Moreover, HIF-1α promoter is located at an NF-κB site, and NF-κB activation promotes HIF-1α expression under hypoxic conditions (Belaiba et al. 2007; Li et al. 2018).

Ginsenoside metabolite compound K (20-*O*-d-glucopyranosyl-20(S)-protopanaxadiol, CK) is the active metabolite of ginsenoside following oral administration and the major form in which ginseng is absorbed *in vivo* (Hasegawa 2004). It has various pharmacological activities, such as anti-inflammatory and immunomodulatory effects. Our earlier findings showed mifepristone, the inhibitor of glucocorticoid receptor (GR), reversed the therapeutic effect of CK on FLS abnormal activation and joint protection (Wang et al. 2016). However, few studies have reported on the molecular mechanism of CK regulating FLS glycolysis. Therefore, we investigated the effect of CK on aberrant activation and glycolysis of FLS through the GR/NF-κB/HIF-1α pathway. This study provides a new perspective on the anti-inflammatory mechanism of CK.

## Materials and methods

### Animals

Male Sprague–Dawley (SD) rats (150 ± 20 g) were purchased from the Experimental Animal Center of Bengbu Medical College. All rats were kept in a controlled environment with a constant temperature (24 ± 2 °C) and humidity (50 ± 10%), a 12 h light/dark cycle, and unrestricted access to food and drink. Before the experiments, rats were habituated to their new surroundings for three days. All experimental procedures followed Bengbu Medical College’s Guidelines for Animal Experiments and were approved by the Ethics Review Committee for Animal Experimentation (Certificate No. 2019-017).

### Drugs and reagents

CK (no. S141001) was transformed from ginsenosides by microbial fermentation technology in Zhejiang Haizheng Pharmaceutical Co., Ltd. (Taizhou, China). Dulbecco’s modified Eagle’s medium (DMEM) was purchased from Gibco (no. C11965500BT, CA, USA), and fetal bovine serum (FBS; no. 086-150) was purchased from WISENT Inc. (Montreal, Canada). The lactate assay kit (no. A019-2-1) was purchased from the Nanjing Jiancheng Bioengineering Institute (Nanjing, China), and the glucose assay kit (no. 361510) was purchased from Shanghai Rongsheng Biological Pharmaceutical Co., Ltd. Small interfering RNAs (siRNAs) for GR, the control siRNA, and plasmids for GR were obtained from Gene Pharma (Suzhou, China).

### Induction of arthritis and treatment

Complete Freund’s adjuvant (CFA) was obtained, as previously reported (Wang et al. 2020). To induce adjuvant arthritis (AA) model, 0.1 mL CFA (10 mg/mL) was injected into the right hind metatarsal foot pad of rats. The control group was injected with normal saline. After 15 days of arthritis induction, rats were randomly separated into four groups (*n* = 10 in each group) based on two systemic manifestations: degree of paw swelling degree and arthritis index score after inflammation. The groups consisted of the normal group, AA group, CK group (80 mg/kg/day intragastric (i.g.), for 18 days), and dexamethasone (Dex) group (1 mg/kg intraperitoneally (i.p.), every three days for 18 days). Before administration, CK was suspended in 0.5% sodium carboxymethylcellulose (CMC-Na) (Liu et al. 2014). The untreated normal and AA groups received vehicle (0.5% CMC-Na) in the same manner daily for 18 days.

For the preparation and *in vivo* dose selection of CK, we referred to studies on CK in the treatment of AA model as well as previous studies by our group. The mode of administration of most traditional Chinese medicines is oral. Our previous studies showed that CK (i.g.) significantly alleviated paw swelling and restored the histopathological change of joint at the doses of 80 and 160 mg/kg in AA rats; intragastrically administered CK (80 and 160 mg/kg) also significantly alleviated carrageenan-induced paw oedema and significantly increased inflammatory pain threshold in rats (Wu et al. 2014; Wang et al. 2016; Chen et al. 2019). Therefore, we selected 80 mg/kg as our experimental dosing *in vivo*. In addition, oral administration of CK has also shown good relief in other disease models such as airway inflammation, intestinal inflammation, and diabetic nephropathy (Wang et al. 2015; Song et al. 2018; Lee et al. 2022). In contrast, Dex was chosen as the positive control drug in this study because of the similarity in chemical structure between CK and Dex. The *in vivo* administration and dose of Dex were based on the studies of Wang et al. (2020) and Zeng et al. (2013), who demonstrated that this dose significantly alleviated the severity of the joint deformity in rats. An intraperitoneal injection of this dose was similar to the therapeutic effect of 0.9 mg/kg methotrexate (once weekly, i.g.).

### Hind paw swelling evaluation

An electronic water plethysmograph (YLS-7C, Anhui Zhenghua Biotechnology Co., Ltd., China) was used to assess the extent of the left (non-injected) hind paw swelling before and every three days after CFA injection. The volume change in each rat paw over time was calculated as the volume after CFA injection minus the volume before injection (Zhou et al. 2019).

### Systemic arthritis score

After immunization, the systemic manifestations of inflammation in the ear, nose, tail, and paw of rats were observed and scored every 3 days using a macroscopic scoring system: 0 = no nodule and redness in the ear, nose, and tail, no paw swelling or redness; 1 = nodule and redness in one ear, nose or tail, one front or hind paw swelling and redness; 2 = nodule and redness in both ears, two front or two hind paws swelling and redness (Wang et al. 2011).

### Radiological examination

The paw and ankle joints of rats in each group were assessed by radiography using a Bruker *in vivo* Multispectral FX PRO (Bruker Corporation, MA, USA) 33 days after immunization to determine the degree of the left (non-injected) hind paw swelling.

### Histological examination

On day 33 after the CFA injection, rats were sacrificed. Synovial tissues and spleens were preserved in 4% paraformaldehyde. The samples were dehydrated using an ethanol gradient series before being embedded in paraffin and sectioned. Histological alterations were examined by staining sections with hematoxylin and eosin (H&E) and observing them under a light microscope. Changes in the spleen were scored as previously described (Li et al. 2012).

### Macrophage phagocytosis analysis

After the rats were sacrificed on day 33, 2 mL of pre-chilled sterile phosphate-buffered saline (PBS) was injected into the abdominal cavity under aseptic conditions and the abdomen was massaged to accumulate the macrophages. The suspension of macrophages was withdrawn and centrifuged (12,500 rpm, 10 min). DMEM containing 10% FBS was used to resuspend cells to a six-well plate and incubate for 4 h. After washing with PBS three times to remove the supernatant, the adherent cells were purified macrophages.

After obtaining macrophages by the above method, the cells were resuspended in 96-well plates at a density of 2 × 10^5^ cells/well. After 24 h, PBS was used to remove unadhered cells. Then, 0.05% neutral red reagent was added and incubation was continued for 3 h. The supernatant was discarded, washed twice with PBS, and the phagocytosis of macrophages was observed under the microscope.

### Immunohistochemistry analysis

Paraffin slices of synovial tissue were prepared and subjected to xylene dewaxing. After rehydration with an alcohol gradient, sections were incubated with hydrogen peroxide to deactivate endogenous catalases, followed by antigen retrieval. The slices were incubated overnight at 4 °C with primary antibodies against GlUT1, PKM1, PKM2, and HK2. On the second day, slices were washed three times and incubated with a secondary antibody for 1 h at room temperature. Colour development of all slices was conducted by diaminobenzidine chromogenic reaction and hematoxylin counterstaining. Images were captured using a microscope.

### Cell culture and treatment

The synovial tissues of rats from all groups were isolated and minced to an adequate size under sterile conditions. The tissues were then transferred to the bottom of a culture flask that had been supplemented with a complete medium (DMEM: FBS = 4:1), penicillin (200 U/mL), and streptomycin (200 ng/mL). When the spindle-shaped synoviocytes migrated out from the tissue blocks and grew to approximately 80% confluency, we removed the tissue and cultured the FLS. Cells from passages 3 to 5 were used for subsequent experiments.

### Cell proliferation assays

The viability rate of FLS was monitored using the Cell Counting Kit-8 (CCK-8) assay kit (BS350A, Biosharp, China). Briefly, cells from the rats of each group (the normal group, AA group, CK group, and Dex group) were trypsinized and resuspended in 96-well plates (5 × 10^3^ cells/well) with 100 μL DMEM containing 5% FBS and then cultured in a constant-temperature incubator (37 °C, 5% CO_2_) for 24 h. CCK-8 solution (10 μL) was added to the plate under sterile conditions, and cells were incubated for a further 2 h. The optical density (OD) was measured using a Synergy HTX Multi-Mode Microplate Reader: Fluorescence-Luminescence (Agilent Technologies, CA, USA) at 450 nm.

For detecting the appropriate concentration of TNF-α to stimulated FLS *in vitro*, TNF-α was added to FLS cultured in 96-well plates to make final concentrations of 10, 20, 30, and 40 ng/mL and incubated for 48 h. A negative control group without TNF-α and a blank group without cells were also set up. The operation of the subsequent absorbance measurement was the same as above. Cell viability rate = (OD of experimental group – OD of blank group)/(OD of negative control group – OD of blank group) × 100%.

For detecting the appropriate concentration of CK to treat FLS *in vitro*, CK was added to FLS cultured in 96-well plates to make final concentrations of 10, 20, 30, 40, and 50 μM and incubated for 48 h. A TNF-α group, a negative control group without CK, and a blank group without cells were also set up. The subsequent operation to detect the absorbance was the same as above. Cell survival rate = (OD of CK group – OD of blank group)/(OD of TNF-α group – OD of blank group) × 100%.

### Flow cytometry assays

An Annexin V/PI apoptosis detection kit (BB-4101, Bestbio, P.R. China) was used to assess cell apoptosis. Briefly, FLS were harvested and suspended in 300 µL of 1 × binding buffer at a concentration of 1 × 10^6^ cells/mL and then incubated in the dark with 2 µL Annexin V-FITC for 15 min and 3 µL PI for 5 min. The samples were immediately detected on a flow cytometer (FACSVerse, BD Bioscience, NJ, USA), and the data were analyzed using FlowJo software (version 7.6).

### Wound healing assay for horizontal migration

The bottom of a 6-well plate was marked with a marker to indicate the same location point for later in the experiment. FLS from each group were resuspended in 6-well plates (5 × 10^5^ cells/well) and incubated in 2 mL of DMEM containing 5% FBS until the cells reached approximately 90% confluency. We then used a 200 μL pipette tip to scratch cell monolayers and removed cell debris by washing twice with sterile PBS. Images were recorded immediately after washing. After 24 h, images of the same location were captured again under an inverted microscope (IX73, Olympus Corporation, Tokyo, Japan), and the scratch area was analyzed using ImageJ software (version 1.8.0.172).

### Transwell assays for vertical migration and invasion

Transwell filter chambers (Corning Inc., NY, USA) were used to analyze the differences in vertical migration and invasion of cells in each group. For the migration assay, cells were trypsinized and resuspended in 200 μL of DMEM at the upper level of a transwell chamber (2 × 10^4^ cells/well), and 600 μL complete medium (DMEM: FBS = 4:1) was added to the bottom level. After 24 h, cells on the outer surface were fixed with 4% paraformaldehyde for 30 min, and non-migrated cells on the inner surface were removed using a cotton swab. Finally, cells were stained with crystal violet for 15 min and counted by selecting 5 random fields of view using an inverted microscope. For the invasion assay, 100 μL of Matrigel (BD Biosciences) was pre-coated evenly on the membrane of the upper chamber, otherwise, the experiment was the same as the migration assay.

### Determination of glucose and lactate concentrations

FLS from each group were cultured as described above. The growth medium of the cells was then collected and tested for lactate and glucose concentrations using a lactate test kit and a glucose test kit, respectively, following the manufacturer’s instructions. Briefly, the supernatant of the culture medium was collected from each group of cells. The supernatant (100 µL) was mixed with 1000 µL glucose detection working solution; 20 µL of supernatant was mixed with 1200 µL lactate detection working solution. The OD at 505 nm or 530 nm was measured in a multi-mode microplate reader, respectively.

### Knockdown or overexpression of GR

For the transfection of GRsiRNA, transient transfections of siRNAs (50 nM) for GR or the nontarget control were performed in normal cultured FLS by Lipofectamine 2000 (Invitrogen) for 6 h. Subsequently, we replaced the fresh culture medium and conducted drug intervention according to the following groups and incubated for 48 h: the control group, TNF-α Group, TNF-α + CK group, TNF-α + CK + siNC (Negative control siRNA) group, and TNF-α + CK + siGR (GR siRNA) group.

Similarly, for the transfection of GR overexpression plasmid, GR was subcloned into pEX-3 (pGCMV/MCS/Neo) plasmid vector and 5 μg plasmid DNA was transfected by Lipofectamine 2000. Subsequently, we replaced the fresh culture medium and conducted drug intervention according to the following groups and incubated for 48 h: the control group, TNF-α Group, TNF-α + CK group, TNF-α + CK + vector (Negative control vector) group, and TNF-α + CK + oeGR (Over-expression of GR) group.

### Western blot analysis

FLS of each group were collected and lysed. All cell lysates were centrifuged to obtain supernatants, and the total protein concentrations were calculated using a BCA assay kit (P0012, Beyotime Biotechnology, China). After boiling in loading buffer, equal amounts (50 μg) of protein extracts were loaded on 8% SDS-PAGE gel for electrophoresis and imprinted onto polyvinylidene fluoride (PVDF) membranes (MilliporeSigma, MA, USA). The membranes were blocked with 5% skim milk for 4 h and washed three times with PBST (phosphate buffered saline + 0.1% Tween-20). Finally, membranes were incubated with primary antibodies (GLUT1, CST, 73015; HK2, CST, 2867; PKM2, CST, 4053; PKM1, CST, 7067; GR, CST, 12041; p-GR, CST, 4161; P65, CST, 8242; p-P65, CST, 3033; IκB, CST, 4812; p- IκB, CST, 9246; HIF-1α, CST, 14179; and β-actin, Proteintech, 60008-1-Ig) overnight at 4 °C. The membranes were conjugated with secondary antibodies (anti-mouse IgG, HRP-linked antibody, CST, 7076; and anti-rabbit IgG, HRP-linked antibody, CST, 7074) at room temperature for 2 h on the second day. After washing with PBST, the blots were analyzed using a chemiluminescence gel imaging system (Fusion FX Spectra, Vilber, Marne-la-Vallée, France). Images were quantified using ImageJ software.

### Immunofluorescence analysis

A density of 3 × 10^4^ cells/well of cells was incubated for 24 h in a small laser confocal dish, which was followed by fixation for 20 min (4% paraformaldehyde), permeabilization for 30 min (0.5% Triton X-100), and blocking for 30 min (10% bovine serum albumin) at room temperature, respectively. These were washed twice with PBS between each step. Finally, the primary antibody was added to all samples and incubated at 4 °C overnight. The next day, after incubation with the corresponding secondary antibody for 2 h, all samples were stained with DAPI for 10 min. Images were captured using a fluorescence microscope (Observer Z1, ZEISS, Germany).

For paraffin Immunofluorescence, paraffin slices of synovial tissue were deparaffinized and followed by antigen retrieval. They were then incubated with 3% H_2_O_2_ for 25 min and 3% BSA for 30 min at room temperature. Vimentin primary antibody (GB12192, Servicebio) was added to all slices and incubated at 4 °C overnight. The next day, the corresponding secondary antibody was added, and the slices were incubated at room temperature for 50 min in dark conditions. Then GR primary antibody (12041, CST) was incubated in the same way. Finally, after the DAPI counterstain in the nucleus, images were collected by fluorescent microscopy.

### Statistical analysis

Data in the figures and text are presented as mean ± standard deviation (SD). Differences between groups were determined using one-way analysis of variance (ANOVA) with GraphPad software (version 8.3.0, GraphPad, CA, USA), and a *P* value < 0.05 was considered significant.

## Results

### CK ameliorates the severity of arthritis in AA rats

**Figure 1.**
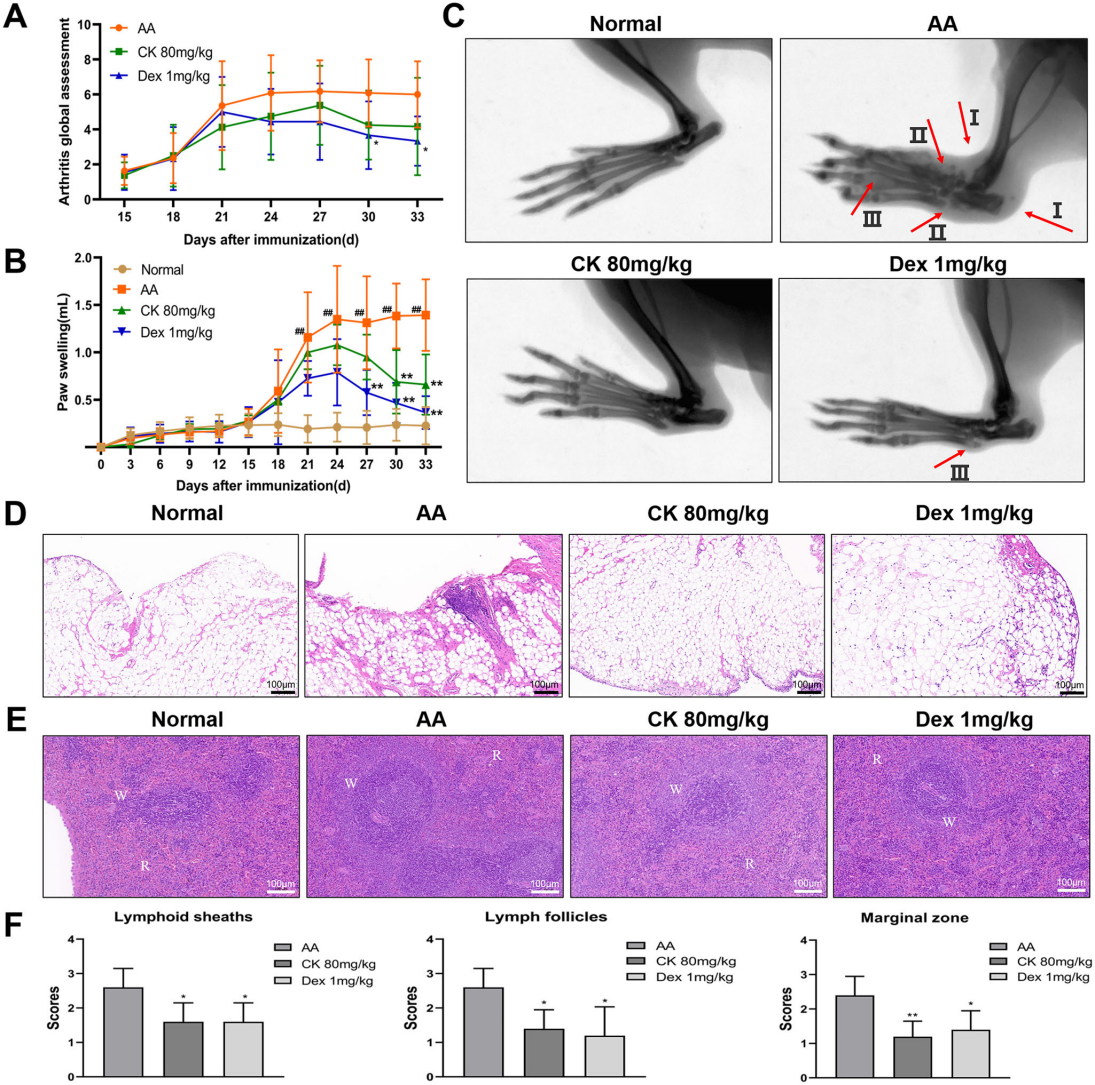
CK alleviates the inflammation of AA rats. (A) Systemic inflammation score. (B) Hind paw swelling of rats in each group. (C) The secondary hind paws were examined by X-ray imaging. I: edema, II: disfiguration, III: bony local density reduction. (D) Synovial tissues were assessed by H&E staining (100 × magnification). (E) Photomicrograph of the spleen (100 × magnification, H&E staining). W: white pulp, R: red pulp. (F) a scoring system that included periarteriolar lymphoid sheaths, lymph follicles, and the marginal zone was used to evaluate the pathological changes in the spleen. Data were from ten rats for each group and expressed as mean ± SD. *p < 0.05, **p < 0.01 vs AA group; ^#^*p* < 0.05, ^##^*p* < 0.01 vs normal group.

During the entire course of inflammation, hind paw swelling and systemic inflammation were monitored and scored every three days. The results showed that the systemic score of the AA group gradually increased until day 24 and inclined to stabilize (Figure 1(A)). Compared with the AA group, swelling of the hind paws was significantly reduced in the CK (80 mg/kg) and Dex (1 mg/kg) treatment groups, specifically from days 27 to 33 (Figure 1(B)). In rheumatoid joints, the synovium transforms into an invasive proliferative tissue mass known as pannus, which can destroy the structure of the joint tissue and eventually lead to cartilage and bone destruction (Nygaard and Firestein 2020). To evaluate the changes in the bone of rats, the secondary hind paws in each group were examined by radiography. The results indicated that the AA group showed obvious oedema, disfiguration, and bony local density reduction. However, the CK (80 mg/kg) group displayed relief from these symptoms (Figure 1(C)).

Generally, a thin layer of synoviocytes in the joints of normal rats is observed. However, as RA disease progresses, synoviocytes begin to proliferate and are infiltrated by inflammatory cells. On one hand, synovial tissue pathology showed that the proliferation of synoviocytes in the AA group was increased compared to the normal group; on the other hand, compared with the AA group, CK (80 mg/kg) group displayed a reduction (Figure 1(D)). White and red pulps were the main components of the spleen in normal rats. The white pulp is an aggregation area of lymphocytes and consists of the central artery, the lymphoid sheath around the artery, and the lymph nodes. When inflammation develops, the white pulp proliferates to various degrees. The results of H&E staining showed that white pulp hyperplasia of AA rats was characterized by an increase in the lymphocyte aggregation area, thickening of the lymphoid sheath around the artery, an increased number of lymph nodes, and widening of the marginal zone. However, these symptoms were significantly mitigated after treatment with CK (Figure 1(E and F)).

### CK alleviates inflammation by regulating the activation and polarization of macrophages

**Figure 2.**
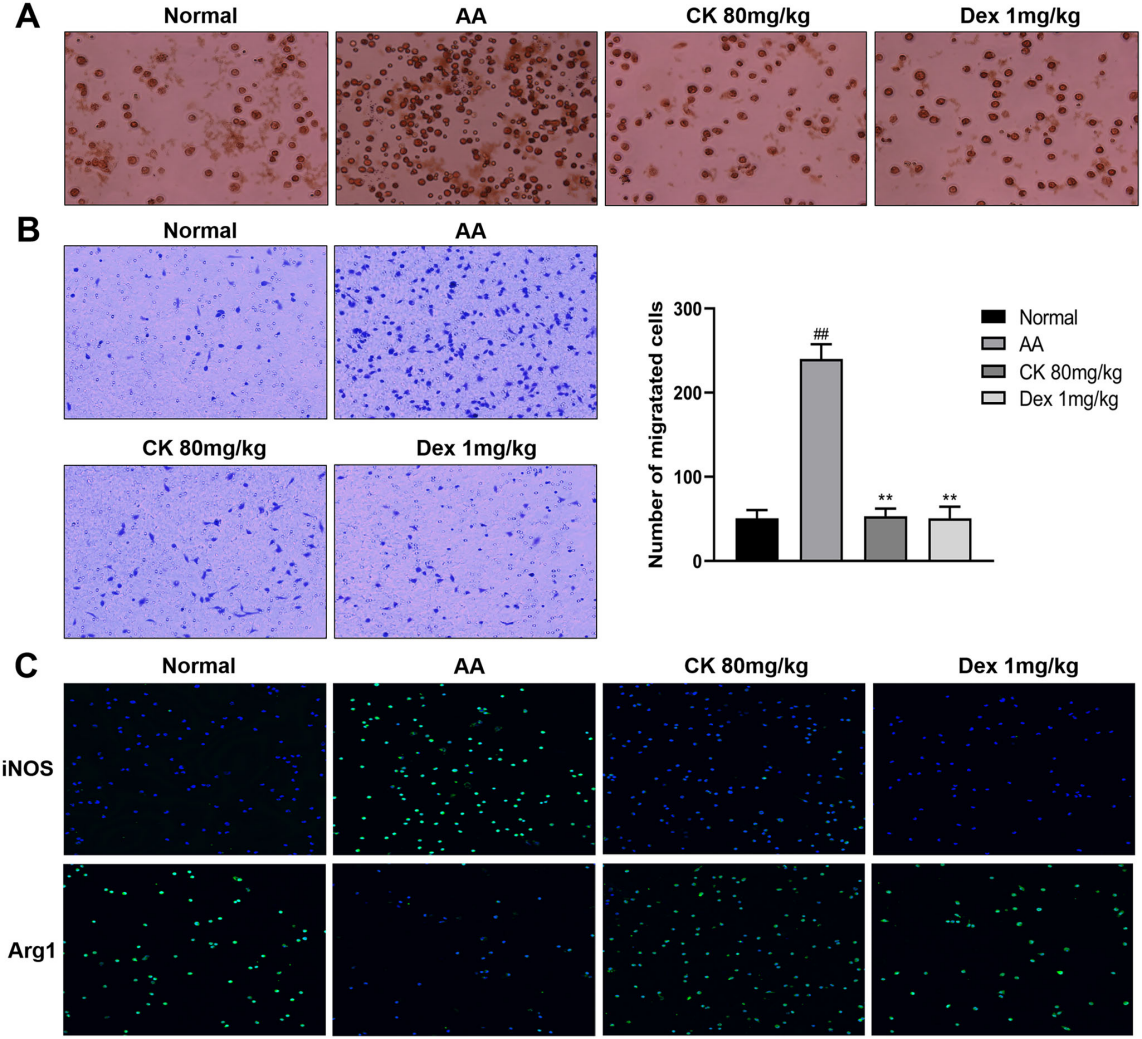
CK regulates the function of macrophages in AA rats. (A) Representative images of macrophage phagocytosis assay (200 × magnification). (B) Migration of macrophages and quantitative analysis (200 × magnification). (C) The expression of Arg1 and iNOS in macrophages of each group was observed under a fluorescent microscope (200 × magnification). Data were from three independent experiments and expressed as mean ± SD. **p < 0.01 vs. AA group; ^##^*p* < 0.01 vs. normal group.

Macrophages are important participants in immune regulation. Under the stimulation of inflammation, macrophages can activate and differentiate into M1 (pro-inflammatory) polarized phenotype. Activated macrophages can recruit immune cells and FLS to promote synovial inflammation by producing a lot of pro-inflammatory factors and cytokines. However, this chronic inflammatory environment can aggravate the activation and polarization of macrophages to form a vicious circle (Zhao et al. 2021). Therefore, we extracted rat peritoneal macrophages and found that the CK treatment group inhibited the phagocytosis and migration of macrophages (Figure 2(A and B)). In addition, CK (80 mg/kg) also up-regulated the level of Arg1, a macrophage marker of M2 (anti-inflammatory phenotype), and down-regulated the level of iNOS, a macrophage marker of M1 (Figure 2(C)). These results indicated that CK can alleviate inflammation by inhibiting the activation of macrophages and correcting the polarization of macrophages.

### CK promotes apoptosis and inhibits the proliferation of AA FLS

**Figure 3.**
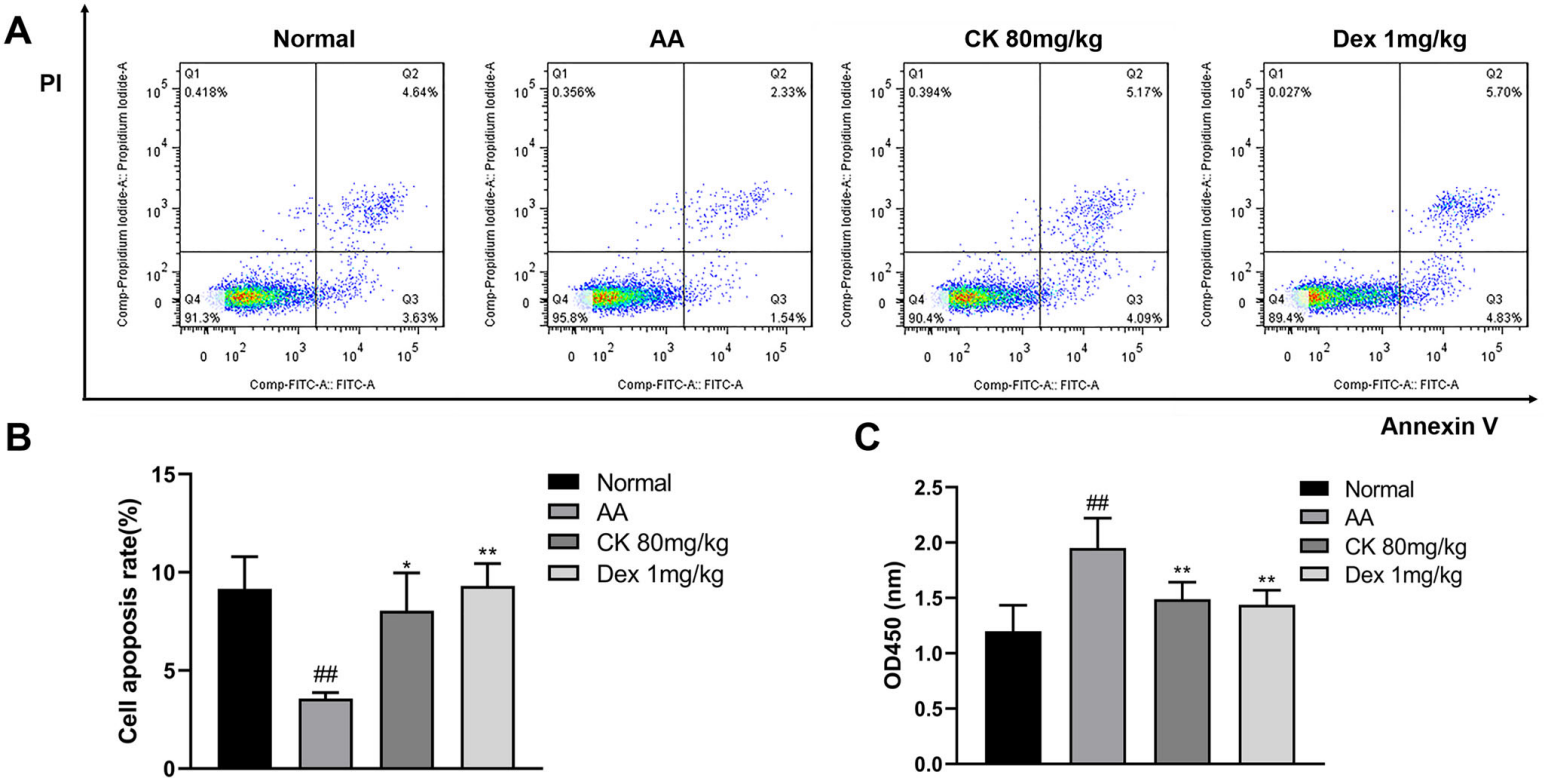
Changes in the apoptosis and proliferation of FLS in each group. (A) Representative pictures of apoptosis and (B) the apoptosis rate were presented as a histogram. (C) The proliferation of FLS was assessed by CCK-8. Data were from three independent experiments and expressed as mean ± SD. *p < 0.05, **p < 0.01 vs. AA group; ^##^*p* < 0.01 vs. normal group.

In RA, FLS shows excessive proliferation and inhibition of the apoptosis pathway. The disruption of the balance between proliferation and apoptosis of FLS is one of the main reasons for their extensive proliferation (Zhou et al. 2021). Next, we obtained FLS, as described above, and observed cell proliferation and apoptosis in each group. The results showed that treatment with CK significantly promoted apoptosis (Figure 3(A and B)) and inhibited proliferation (Figure 3(C)) of the FLS compared with that in the AA group.

### CK suppresses AA FLS migration and invasion

**Figure 4.**
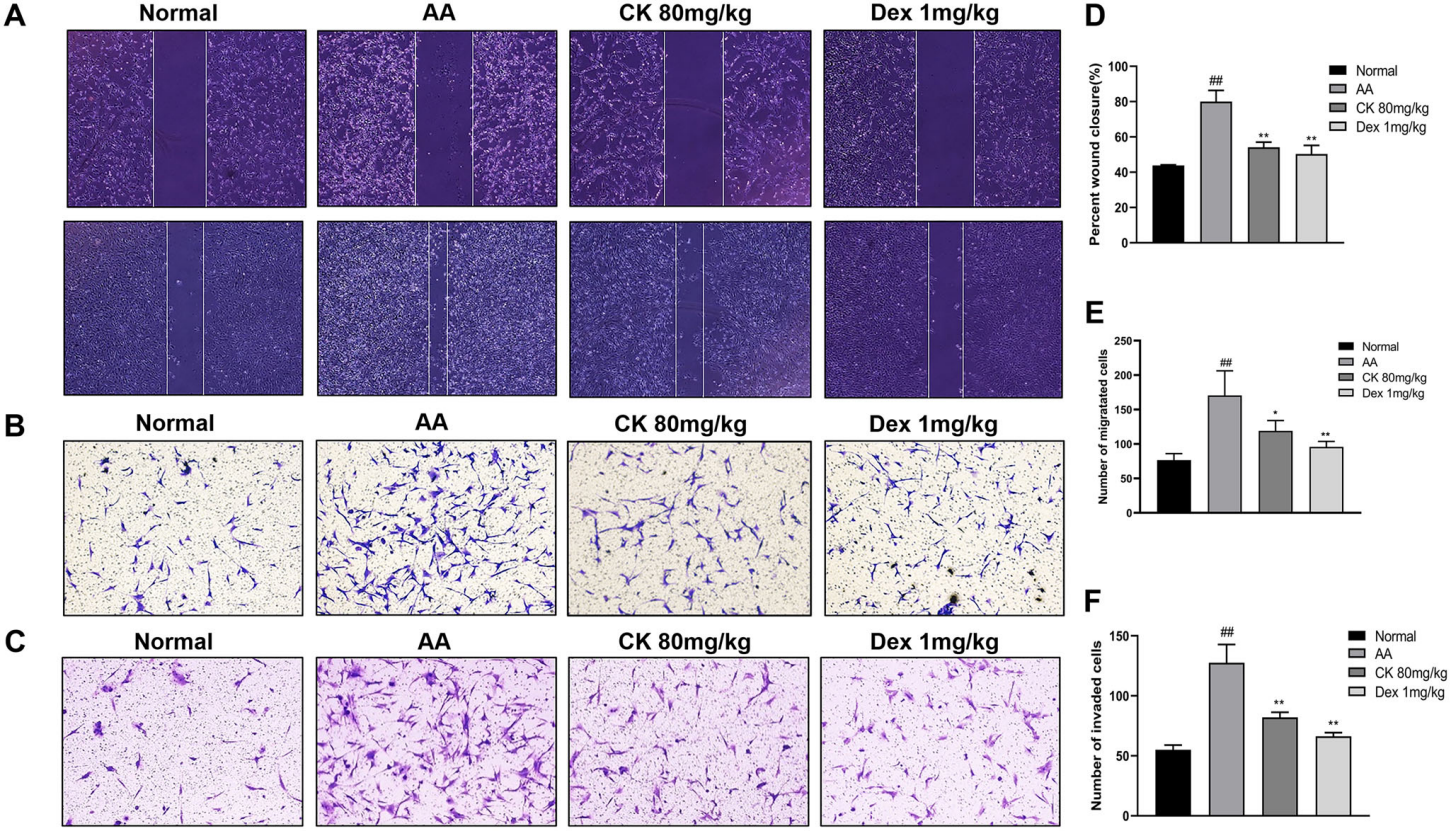
The migration and invasion in FLS with the treatment of CK. Representative images show (A) wound healing (40 × magnification), (B) migration (100 × magnification), and (C) invasion (100 × magnification) in different groups. Quantitative analysis of (D) wound healing, (E) migration, and (F) invasion. Data were from three independent experiments and expressed as mean ± SD. *p < 0.05, **p < 0.01 vs. AA group; ^##^*p* < 0.01 vs. normal group.

As the main cell type of synovial cells, FLS exhibit a range of tumour-like biological behaviours including massive proliferation, migration, and invasion (Mor et al. 2005). Next, to evaluate the effect of CK on the aggressive invasive ability of AA FLS, we use wound healing assay for horizontal migration and transwell assays for vertical migration and invasion. According to our findings, CK substantially reduced the migration and invasion of AA FLS (Figure 4)

### CK inhibits the glycolysis activity of AA FLS

**Figure 5.**
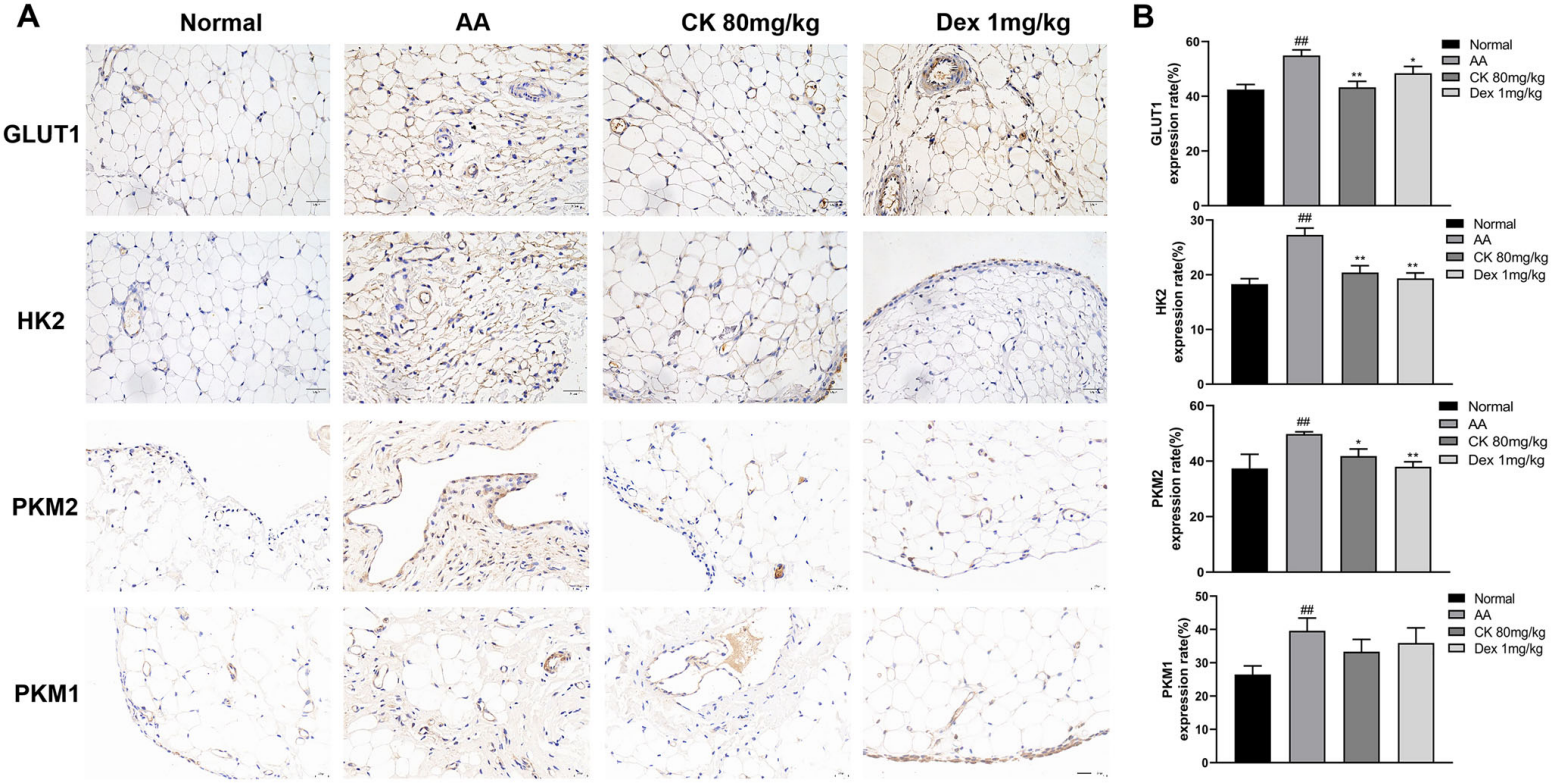
Representative images showing the expression of GLUT1, HK2, PKM2, and PKM1 in (A) synovium. (B) Bar graphs were used to quantify the expression of GLUT1, HK2, PKM2, and PKM1. Data were from three independent experiments and expressed as mean ± SD. *p < 0.05, **p < 0.01 vs. AA group; ^##^*p* < 0.01 vs. normal group.

**Figure 6.**
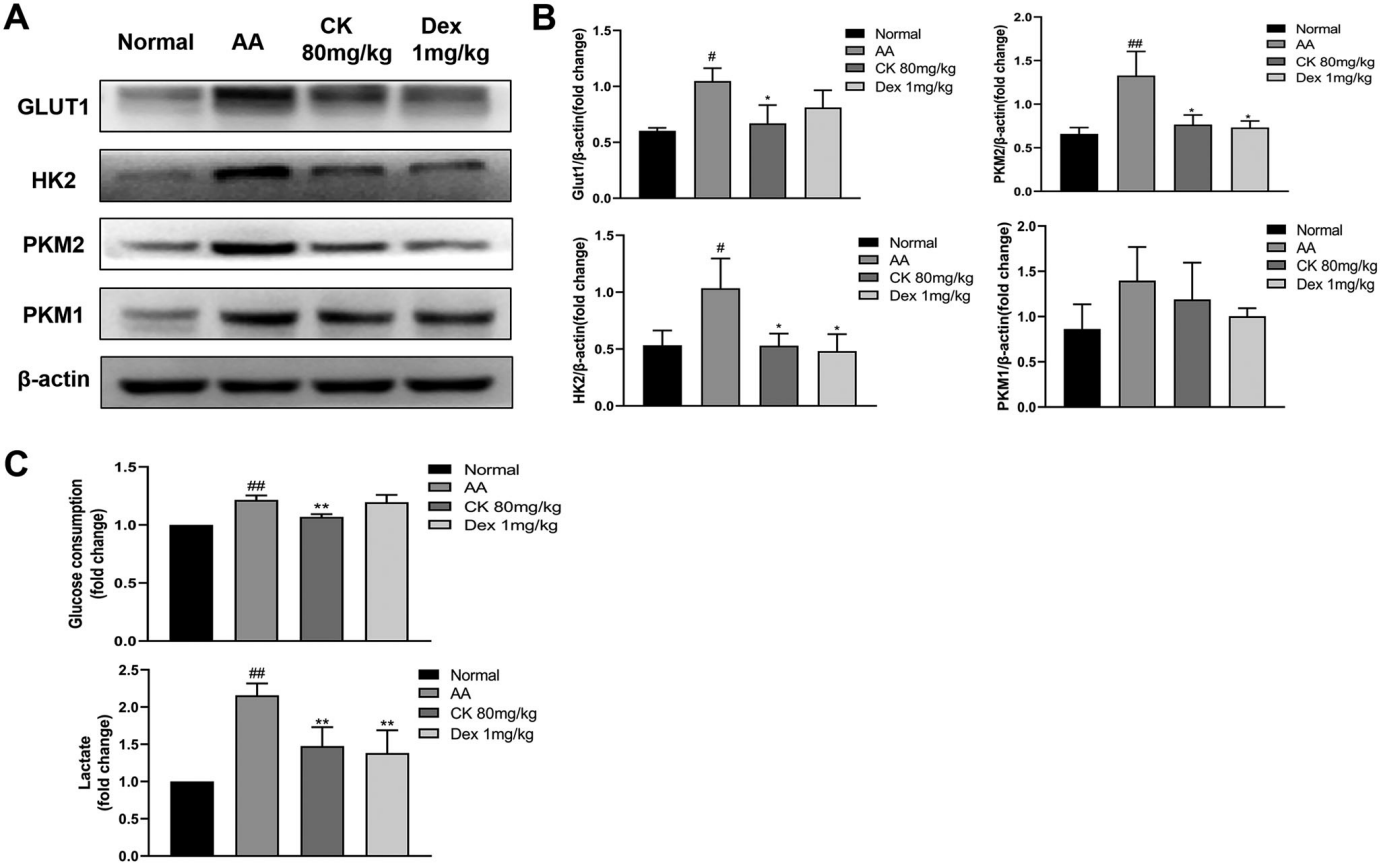
CK effectively affects the glycolysis of FLS. (A and B) the levels of GLUT1, HK2, PKM2, and PKM1 of FLS in each group were detected by Western Blot. (C) Levels of glucose consumption and lactate excretion. Data were from three independent experiments and expressed as mean ± SD. *p < 0.05, **p < 0.01 vs. AA group; ^#^*p* < 0.05, ^##^*p* < 0.01 vs. normal group.

Metabolomic studies have indicated that the abnormal activation of glycolysis in FLS is an important mechanism for the early onset of RA, which includes increased glucose consumption and lactate export and increased expression of glucose transporter 1 (GLUT1), hexokinase 2 (HK2), and pyruvate kinase M2 (PKM2) (Masoumi et al. 2020). Therefore, we investigated the effects of CK on these changes. The immunohistochemistry and western blotting showed that CK downregulated the expression of the glycolytic enzymes GLUT1, HK2, PKM2, and PKM1 in comparison with that in the AA group (Figures 5 and 6(A and B)). In addition, CK also significantly reduced the glucose consumption and lactate export of AA FLS (Figure 6(C)). These results suggested that CK can inhibit the abnormal glycolytic activity of FLS.

### CK inhibits the NF‑κB/HIF-1α signalling pathway by activating GR

**Figure 7.**
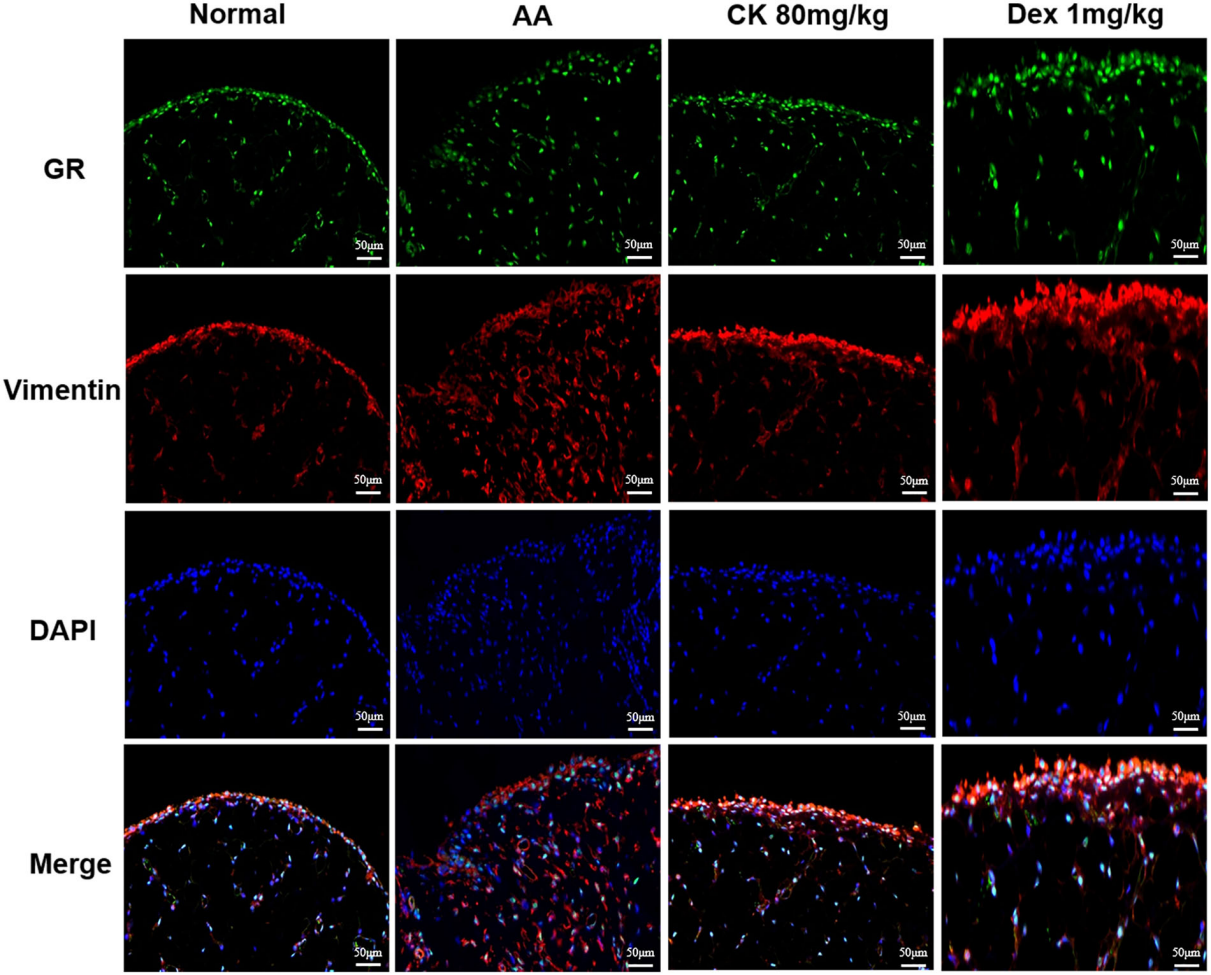
Double-label immunofluorescence (paraffin-slides) showing the nuclear translocation of GR in synovial tissues (200 × magnification). IF red and green were conducted to detect vimentin and GR.

**Figure 8.**
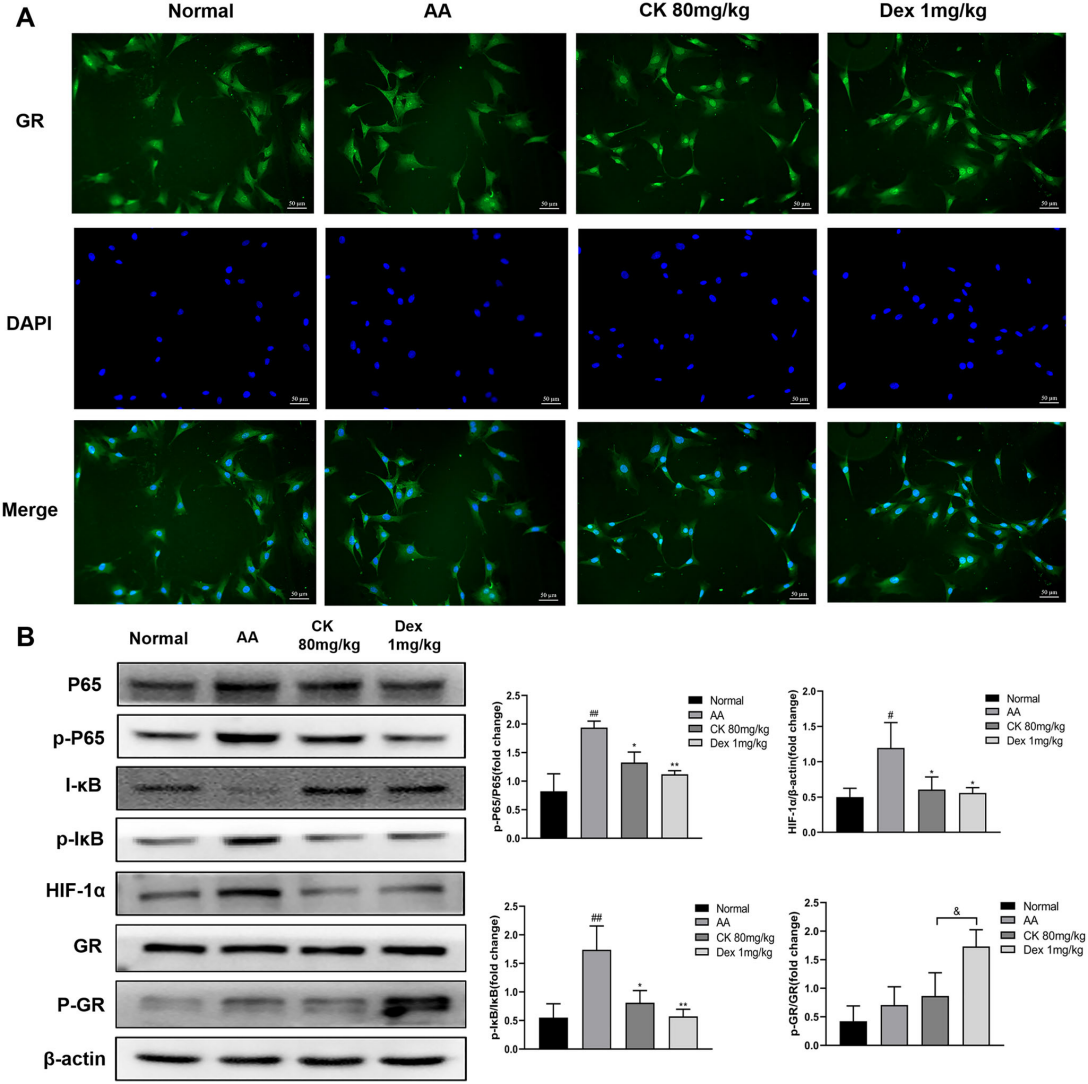
Effects of CK on GR/NF-κB/HIF-1α pathway in FLS. (A) Representative fluorescence microscope pictures showing the nuclear translocation of GR (200 × magnification). (B) The expression of HIF-1α, P65, p-P65, IκB, p-IκB, GR, p-GR, and β-actin in FLS, and bar graphs were used for quantitative evaluation. Data were from three independent experiments and expressed as mean ± SD. *p < 0.05, **p < 0.01 vs. AA group; ^#^*p* < 0.05, ^##^*p* < 0.01 vs. normal group; ^&^*p* < 0.05.

The GR/NF-κB/HIF-1α pathway was examined to further clarify the mechanism by which CK inhibits glycolysis to alleviate AA rats. We first observed the synovial tissues by double-labelling immunofluorescence (paraffin-slides). IF red was conducted to detect vimentin which could dye onto the FLS. IF green was conducted to detect GR. The result found that the nuclear translocation of GR decreased in the AA group, while CK triggered the nuclear translocation of GR (Figure 7). Similarly, the same results were found in the FLS immunofluorescence assay (Figure 8(A)). Furthermore, the results of western blotting indicated that the phosphorylation of NF-κB p65 and the expression of HIF-1α in the AA group was significantly up-regulated compared with that in the normal group. However, CK treatment significantly decreased the level of p-P65 and HIF-1α in the AA FLS (Figure 8(B)). The data above imply that CK can regulate the NF-κB/HIF-1α pathway by activating GR. Therefore, we propose to speculate whether CK can regulate the glycolysis of FLS through GR.

**Figure 9.**
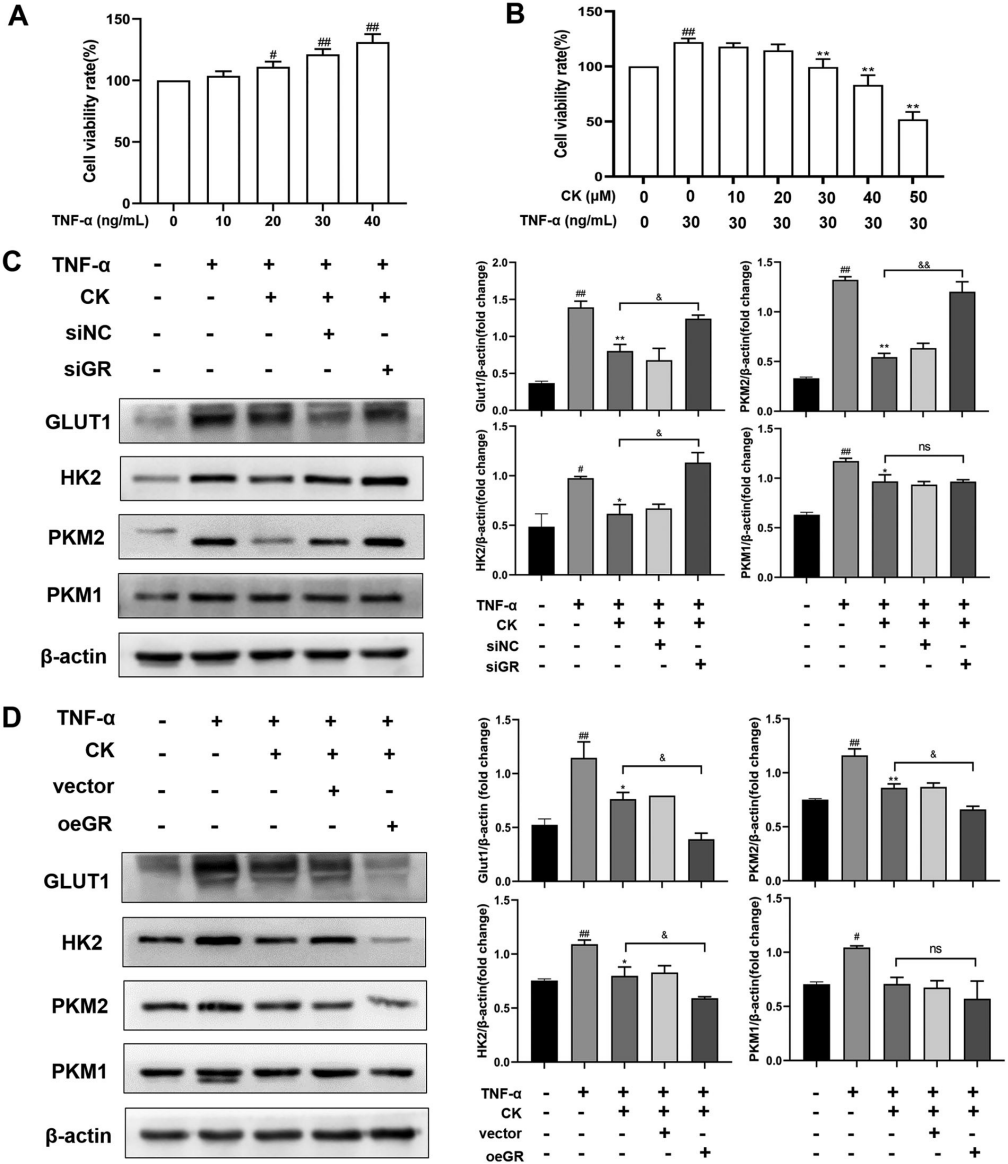
CK inhibits the glycolysis of FLS in a GR-dependent manner. (A) Effects of TNF-α on viability rate of FLS. (B) Effects of CK on viability rate of FLS stimulated by TNF-α. FLS of normal SD rats were transfected for 6 h, and TNF-α (30 ng/ml) and CK (30 μM) were added to the corresponding groups for 48 h. (C) The expression of GLUT1, HK2, PKM2, and PKM1 of FLS were measured after GRsiRNA transfection. (D) The expression of GLUT1, HK2, PKM2, and PKM1 of FLS were measured after transfection with GR overexpression plasmid. Data were from three independent experiments and expressed as mean ± SD. *p < 0.05, **p < 0.01 vs. TNF-α group; ^#^*p* < 0.05, ^##^*p* < 0.01 vs. control group; ^&^*p* < 0.05, ^&&^*p* < 0.01.

Tumour necrosis factor α (TNF-α) has been shown to increase the glycolysis of FLS and transform FLS from static to aggressive cells (de Oliveira et al. 2019). Therefore, we used TNF-α to stimulate FLS of normal SD rats to establish an inflammatory model *in vitro* and knock down or over-express GR to further detect the effects of CK on FLS glycolysis. The results showed that the viability rate of FLS was significantly improved by TNF-α at the concentration of 30 ng/mL compared to that of the control group (Figure 9(A)). Therefore, 30 ng/mL was selected as the concentration of TNF-α to stimulate FLS activation for subsequent experiments. Moreover, the inhibitory effect of CK at 30 µM on FLS proliferation was more significant compared to that of the TNF-α (30 ng/mL) group, and no obvious difference was observed compared to that of the control group (Figure 9(B)). Thus, 30 µM was used as the concentration of CK in the following experiments. As predicted, after the transfection of GRsiRNA, the effect of CK on down-regulating the expression of the glycolytic enzyme in the TNF-α + CK + siGR group weakened compared to that of the TNF-α + CK group (Figure 9(C)). However, this effect was reversed after GR overexpression (Figure 9(D)). These results indicate that CK inhibits FLS abnormal glycolysis and NF-κB/HIF-1α pathway in a GR-dependent manner.

## Discussion

Pharmacokinetic studies have shown that CK, the metabolite of ginsenosides after oral administration, is the primary mediator of ginseng efficacy (Hasegawa 2004). Since CK was isolated from ginsenoside using biotransformation approaches, its anti-inflammatory, antitumor, hepatoprotective, and other pharmacological activities have been gradually explored (Liu et al. 2022). Our data demonstrated that CK therapeutically affects the course of the AA rat model, including inhibition of paw swelling, prevention of bone damage, and improvement of the pathological manifestations of AA in the spleen and synovium. Furthermore, the interaction of diverse immune cells induced by activated M1 macrophages leads to the disorder of the immune microenvironment, which is an important factor to aggravate the course of RA. Our results suggested that CK also inhibited the activation and polarization of macrophages. However, the underlying mechanisms of the anti-inflammatory effects of CK remain unclear.

Notably, as the main cell type of synoviocytes, FLS can promote the production of hyaluronic acid and play an important role in maintaining joint lubrication. However, in RA, fibroblast populations continue to accumulate as pro-apoptotic pathways are suppressed, resulting in dramatic hyperplasia of the lining layer, a 2–3 cell thick lining layer transforming into a mass of pannus tissue. The pannus migrates and invades adjacent joints, eventually leading to the destruction of the cartilage and bone (Nygaard and Firestein 2020). In addition, evidence suggests that the fibroblast population expansion in joints results primarily from the inhibition of pro-apoptotic pathways. The reasons include decreased expression of pro-apoptotic factors such as PTEN and SEN-P1; increased expression of pro-survival factors such as FLIP and SUMO-1; and over-activity of the NF-κB pathway (Korb et al. 2009). Therefore, inhibiting the proliferation, migration, and invasion of FLS is an effective strategy for treating RA. In this investigation, we obtained synovial tissue of rats in each group to culture FLS and observed that CK could significantly inhibit the proliferation, migration, and invasion of AA FLS and significantly promote the apoptosis of AA FLS. These findings support the role of CK in inhibiting FLS aberrant activation.

Recently, increasing studies have revealed that FLS activation has a considerable positive association with metabolic alterations, and therapy options for particular glycolytic enzymes may be targeted. During glycolysis, glucose crosses the cell membrane *via* GLUT1 and is broken down by metabolic enzymes including HK and PKM, to generate lactate. The expression of GLUT1 in RA FLS has been previously demonstrated as substantially higher than that in osteoarthritis (OA) FLS (Garcia-Carbonell et al. 2016; McGarry et al. 2017). In addition to the increased glucose consumption caused by the rapid growth of RA FLS and the high demand for nutrients, the cytokines secreted by RA FLS also contribute significantly to the expression of GLUT1. Therefore, glucose consumption and GLUT1 may be potential biomarkers for RA. Moreover, as the rate-limiting enzyme that catalyzes the first step of glycolytic metabolism, HK2 is specifically overexpressed in RA synovium compared to that in OA samples (Bustamante et al. 2018; Song et al. 2019). Thus, the use of HK2 as a selective target may be safer than the global inhibition of glycolysis. Here, we demonstrate that CK downregulates the expression of GLUT1 and HK2 in synovial tissues and FLS in AA rats and decreases glucose consumption and lactate export. PKM1 and PKM2 are the two isoforms of pyruvate kinase M (the rate-limiting enzyme that catalyzes the final step of glycolysis) (Schormann et al. 2019). PKM2 is generally believed to be marked as overexpressed in tumour cells with high glycolytic activity and is a potential target for tumour therapy (Spencer and Stanton 2019); however, the expression of PKM1 has been controversial (Morita et al. 2018). Our results indicate that the expression of PKM1 and PKM2 is upregulated in AA rats and this situation is alleviated after CK treatment. However, the relationship between PKM1 and PKM2 in cells with high glycolytic activity merits further investigation. Simultaneously, researchers discovered that the enhanced glycolytic activity of RA FLS was closely correlated to the regulation of HIF-1α. HIF-1α has been shown to enhance glycolytic activity *via* the upregulation of HK2, PKM2, and GLUT1 in RA FLS, promoting the production of lactate and FLS invasion, migration, and survival and exacerbating the severity of inflammation (Masoumi et al. 2020; Zheng et al. 2021). Therefore, we assessed HIF-1α levels in AA FLS. The results showed that HIF-1α expression in AA FLS was significantly downregulated after CK treatment.

Glucocorticoids are steroid hormones that show potent anti-inflammatory actions *via* glucocorticoid receptors (GR) and are widely employed in clinical settings. As a transcription factor, the activated GR can activate or suppress the expression of a vast number of target genes through transactivation and transrepression, including many genes responsible for anti-inflammatory and proinflammatory responses. Although glucocorticoids have excellent anti-inflammatory properties, long-term and high-dose usage of glucocorticoids frequently result in osteoporosis and a variety of other side effects, which are mostly due to GR transactivation (Sundahl et al. 2015). In our results, radiography analysis of rat joints demonstrated that dexamethasone (Dex), a traditional glucocorticoid, reduced bone density of local joints despite being anti-inflammatory, whereas CK had both anti-inflammatory and bone-protective effects.

Our previous study showed that the role of CK in alleviating arthritis was related to GR (Wang et al. 2016); hence, we decided to further explore whether the role of CK in regulating glycolysis is mediated by GR. We used immunofluorescence to observe GR nuclear translocation in synovial tissue and FLS of rats in each group because the nuclear translocation of GR is a key indicator of its activation. The findings revealed that nuclear translocation of GR was shown in both the CK and Dex groups, indicating that the inhibition of glycolysis by CK was very likely mediated by GR. Subsequently, to further verify this hypothesis, we knocked down and overexpressed GR to detect the expression of glycolytic enzymes in FLS stimulated by TNF-α again. As predicted, after GR knockdown or overexpression, the inhibition of CK on glycolytic enzymes was weakened or enhanced, respectively. These findings provide more favourable evidence for the close relationship between CK and GR. Furthermore, the expression of phosphorylated GR (p-GR) is another essential indicator of GR transactivation (De Bosscher et al. 2005). Our results showed that compared with Dex group, the expression of p-GR in the CK group was lower, indicating that CK and GR may function more through transrepression rather than transactivation and that CK has fewer glucocorticoid-like adverse reactions. Next, we further explored the possibility of CK activating GR to exert transrepression.

After activation, GR is normally transferred to the nucleus directly or indirectly, interfering with the transcriptional activity of other DNA-binding transcription factors, such as NF-κB. NF-κB activation is associated with the pathogenesis of several inflammatory diseases. Generally, NF-κB activation depends on the degradation of its inhibitor (IκB) protein by phosphorylation. The activated NF-κB dimer then binds to target DNA sequences in the nucleus and regulates the transcription of proinflammatory cytokines, growth factors, chemokines, and other target genes, which are important contributors to aggravating the course of RA (Hayden and Ghosh 2008; Taniguchi and Karin 2018). Interestingly, several NF-κB binding sites in the HIF-1α promoter are observed, and activation of NF-κB leads to an increase in HIF-1α expression (Taniguchi and Karin 2018; Korbecki et al. 2021). This indicates that NF-κB and HIF-1α are critical pathways in the regulation of abnormal glycolysis and activation of FLS. We found that CK inhibited the activation of NF-κB and HIF-1α in AA FLS by downregulating the expression of p-P65, p-IκB, and HIF-1α. We speculated that the inhibition of NF-κB and HIF-1α by CK and the inhibition of abnormal glycolysis of FLS might be mediated by the activation of GR. However, the specific interactions among these factors still need to be explored, and we aim to study them in further research.

## Conclusions

The current study suggests that treatment with CK ameliorates the severity of symptoms in adjuvant arthritis rats. Mechanically, CK inhibited the aberrant glycolysis and the NF-κB/HIF-1α pathway of FLS by activating GR. The present report provided experimental evidence for the clinical usage of drugs and potential useful therapeutic targets for rheumatoid arthritis.

## Data Availability

Derived data supporting the findings of this study are available from the corresponding author on request.
